# Baseline malaria prevalence at the targeted pre-elimination districts in Ethiopia

**DOI:** 10.1186/s12889-021-12036-5

**Published:** 2021-11-03

**Authors:** Desalegn Nega, Adugna Abera, Bokretsion Gidey, Sindew Mekasha, Abnet Abebe, Dereje Dillu, Degu Mehari, Gudissa Assefa, Samuel Hailu, Mebrahatom Haile, Kebede Etana, Hiwot Solomon, Gezahagn Tesfaye, Daniel Nigatu, Zelalem Destaw, Berhane Tesfaye, Belendia Serda, Asnakew Yeshiwondim, Assefaw Getachew, Hiwot Teka, Honelegn Nahusenay, Semira Abdelmenan, Hailemariam Reda, Worku Bekele, Ayele Zewdie, Getachew Tollera, Ashenafi Assefa, Geremew Tasew, Adugna Woyessa, Ebba Abate

**Affiliations:** 1 Ethiopian Public Health Institute, Addis Ababa, Ethiopia; 2 Federal Ministry of Health, Addis Ababa, Ethiopia; 3 Central Statistical Agency, Addis Ababa, Ethiopia; 4 Malaria Control and Elimination Partnership in Africa (MACEPA) at PATH, Addis Ababa, Ethiopia; 5 President’s Malaria Initiative (PMI), Addis Ababa, Ethiopia; 6 Addis Continental Institute of Public Health, Addis Ababa, Ethiopia; 7 Clinton Health Access Initiative, Inc. (CHAI), Addis Ababa, Ethiopia; 8 World Health Organization (WHO), Addis Ababa, Ethiopia

**Keywords:** Baseline survey, Malaria epidemiology, Malaria elimination, Ethiopia

## Abstract

**Background:**

Encouraged by the previous success in malaria control and prevention strategies, the Ethiopian ministry of health launched malaria elimination with a stepwise approach by primarily targeting the low-transmission Districts and their adjacent areas/zones in order to shrink the country’s malaria map progressively. Hence, this community survey was conducted to establish baseline malaria information at the preliminary phase of elimination at targeted settings.

**Methods:**

A community-based cross-sectional survey was conducted at 20 malaria-elimination targeted Districts selected from five Regional states and one city administration in Ethiopia. The GPS-enabled smartphones programmed with Open Data Kit were used to enumerate 9326 study households and collect data from 29,993 residents. CareStart™ Malaria *PAN (pLDH)* Rapid Diagnostic Tests (RDTs) were used for blood testing at the field level. Armpit digital thermometers were used to measure axillary temperature.

**Result:**

Overall malaria prevalence by RDTs was 1.17% (339/28973). The prevalence at District levels ranged from 0.0 to 4.7%. The proportion of symptomatic cases (axillary temperature > 37.5^o^c) in the survey was 9.2% (2760/29993). Among the 2510 symptomatic individuals tested with RDTs, only 3.35% (84/2510) were malaria positive. The 75.2% (255/339) of all malaria positives were asymptomatic. Of the total asymptomatic malaria cases, 10.2% (26/255) were under-five children and 89.8% (229/255) were above 5 years of age.

**Conclusion:**

The study shows a decrease in malaria prevalence compared to the reports of previous malaria indicator surveys in the country. The finding can be used as a baseline for measuring the achievement of ongoing malaria elimination efforts. Particularly, the high prevalence of asymptomatic individuals (0.88%) in these transmission settings indicates there may be sustaining hidden transmission. Therefore, active case detection with more sensitive diagnostic techniques is suggested to know more real magnitude of residual malaria in the elimination-targeted areas.

## Introduction

### Background

During the last decade, substantial worldwide progress has been made in controlling malaria worldwide. The magnitude of the progress has led some malaria-endemic countries to consider the possibility of malaria elimination [1–4]. Ethiopia experienced cycles of major malaria epidemics every 5 to 8 years in highland areas, where the last nationwide epidemic (> 4000 malaria deaths) occurred in 2003 and fewer epidemics since 2004 [5]. The apparent decrease of major malaria epidemics within the last decade is a result of the national scale-up of malaria control interventions in Ethiopia [5, 6]. Mass distribution of insecticide-treated bed nets (ITNs), indoor residual spraying (IRS), and adoption of Artemisinin-based combination therapy (ACT) contributed much to the substantial declines in malaria-related deaths and morbidity [5, 7, 8].

In its current national malaria strategic plan 2021–2025 [9] and in its malaria elimination roadmap [10], Ethiopia declared to achieve national-level malaria elimination in 2030, by prioritizing elimination in targeted low transmission settings and preventing the reintroduction of malaria into Districts reporting zero indigenous malaria cases. Since a decrease in malaria burden noted earlier is not uniform, a sub-national elimination must be attempted before nationwide elimination can be attained [10]. Thus, the country aims to eliminate malaria with a stepwise approach by targeting first the low-transmission Districts (Districts below five cases per 1000 people per year) and their adjacent areas/zones to shrink the country’s malaria map progressively [11].

Several malaria epidemiological surveys have been carried out since 2000 in Ethiopia, with malaria prevalence by microscopy declining from 10.4 to 0.5% in 2015 [12–14]. Some of the nationally-representative surveys were the Demographic and Health Survey (DHS) 2000 [15], DHS 2005 [16], a large survey by the Carter Center [17], the Ethiopia Malaria Indicator Survey (EMIS) 2007, MIS 2011, and MIS 2015 [18–20]. The various findings of these surveys have been reported extensively. Most of these surveys assessed key malaria interventions, treatment-seeking behavior, anemia prevalence in children < 5 years of age (U5), malaria prevalence in all age groups, malaria knowledge among women, and indicators of socioeconomic status. The survey results were stratified by Regional states, by altitude, and by Districts. Compared to DHS 2005 [16], findings from MIS 2007, MIS 2011, MIS 2015 reflected the significant effort of the FMOH-led scale-up of malaria prevention and control interventions, with substantial increases in insecticide-treated net (ITN) ownership and use, as well as malaria knowledge**.**

The most recent survey, EMIS 2015, is a large, nationally representative survey of coverage of key malaria control interventions, treatment-seeking behavior, and malaria prevalence. EMIS 2015 also assessed anemia prevalence in children less than five years of age (U5), malaria knowledge among women, and indicators of socioeconomic status. EMIS 2015 similar to the previous EMISs focuses on areas < 2000 m above sea level (ASL). Differently from EMIS 2011, all Regions, including Dire Dawa City Administration and Harar Region, have separate Regional estimates.

The current survey is a baseline survey conducted in settings targeted for malaria elimination in specific geographies in Ethiopia. Baseline malaria information gathered at the pre-elimination phase is essential to understand the community-level transmission and measure the next outcomes towards the elimination goal. Baseline survey results enable programs (or program managers/interested people) to anticipate achievements and changes in the future regarding interventions administered for malaria prevalence reduction in the communities [21]. A baseline survey on malaria epidemiology and intervention coverage at the community level in the elimination-planned Districts would help to know the status in terms of parasite distribution, intervention coverage, and community awareness to malaria and control approaches, and to evaluate the success of activities exerted for elimination. Therefore, this study was conducted to measure the magnitude of *Plasmodium* infections at the community level in the initial phase of malaria elimination in the targeted Districts and Regions in Ethiopia.

## Materials and methods

### Study area and period

**Fig. 1:**
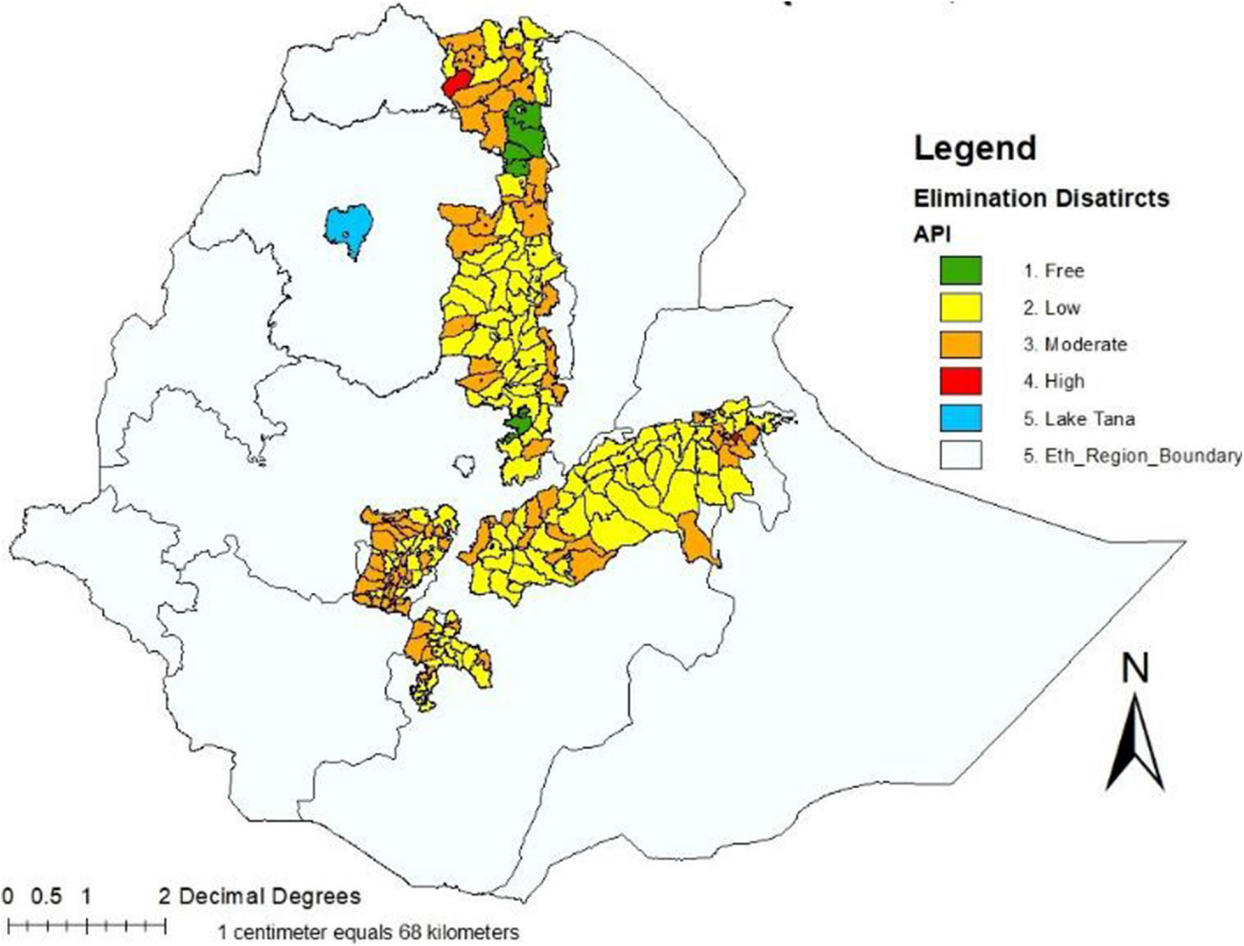
Risk map of Districts by annual parasite incidence, Ethiopia, 2017 (Source FMOH malaria NSP: 2017–2020)

This study was conducted from October to December 2018 in selected Districts targeted for malaria elimination in Ethiopia. Ministry of Health (MOH) launched malaria elimination in 239 Districts with low annual parasites among the five Regional states (Amhara, South Nations and Nationalities, Oromia, Tigray, and Harari Regions) and Dire-Dawa city administration. Ethiopia National Strategic Plan (NSP) 2017–2020 has stratified the country’s malaria situation based on Annual Parasite Incidence (API) per 1000 population. Accordingly, four broad strata have been identified. They are malaria-free (API = 0), low (API= > 0 to ≤10 cases/1000 person-years), moderate (API= > 10 to < 50 cases), and high-transmission (API = ≥50 cases/1000 person-years) strata. The current assessment was commenced in these low-to-moderate malaria transmission Districts in Ethiopia as shown in Fig. 1.

The selected study Districts were from Amhara Region (Berehet, Raya kobo, Bugna, Habru); from Oromia Region (Merti, Sire, Zeway-Dugida, Kersa, Gemechis); from South Ethiopia (Dilla-zuria, Marako, Misrak-Badawacho, Damboya, Dore, Lanfaro); from Tigray Region (Kolla-Tembein, Hawzen, Seharti-samre), and all Districts in Diredawa city administration and Harari Region.

### Study participants

All members of the selected households in the study Districts, those with or without malaria symptoms, aged above 1 year and both sexes were included. Individuals having taken anti-malarial drugs in the past six weeks, non-consenting individuals, children under the age of one year, and absentees at home during data collection were excluded.

### Study design and sampling procedure

The design of the study was cross-sectional. From among the 239 malaria elimination target districts, only 20 districts were selected as study districts proportionally considering the total size of elimination target districts per each region/city administration, intended study objective, higher malaria reports, the average population per district, and available budget. The sample design was framed by Ethiopian Central Statistical Agency (CSA), in collaboration with data management professionals in Ethiopian Public Health Institute (EPHI). The sampling frame used for this survey was a list of malarious kebeles in the selected 20 target districts (domains) selected for the study. In each domain (district), a sample of kebeles with a predetermined sample size was then selected independently with probability proportional to size. A two-stage cluster sampling was used where kebele was the primary sampling unit and the household was the second-stage sampling unit. The design of the survey was cross-sectional and the minimum sample size required for the survey, per domain, was determined using the formula below:$\mathrm{n}={Deft}^2{\left(z\frac{a}{2}\right)}^2\ \left[\frac{p\left(1-p\right){x}^2}{d^2}\right].$

Where, ***n:*** the required minimum sample size per domain; ***α:*** level of significance; ***p:*** prevalence rate; ***d:*** absolute precision (=2*p*ε); ***ε:*** desired relative standard error; ***Deft:*** estimated design effect to account for the two-stage cluster sampling method.

The key indicator taken to determine the sample size was malaria parasite prevalence by microscopy, which was 0.5%, among all age groups residing in malarious areas according to the Ethiopia National Malaria indicator survey 2015. In the above formula, using a relative precision of 0.4%, 0.05 level of significance, design effect of 1.3, and adjusting for the 97% household gross response rate, and taking 20 domains (districts), the total minimum sample size was 9326 households, and 112 kebeles from a total of 406 malarious kebeles in the 20 study districts.

Households were selected with a simple random sampling using EPiSample software programmed in smartphones. To have had a domain-level estimate, precision and design effect were used at district levels. The distribution of the total sample size to the districts; therefore, used a power allocation with an appropriate power value of 0.99 to guarantee a sufficient sample size in small districts.

### Field data collection

During fieldwork, field teams conducted full household listings in each kebele/village using the smartphones programmed with EpiSample software [22] and selected randomly the study households with the support of field technical assistants and guide persons. Questionnaires were programmed into Samsung I9300 S3 Neo smartphones with a capacity of taking global positioning system (GPS) at each household level. After obtaining consent from the study participants, a face-to-face questionnaire interview was held by health officers/nurses. The participants were interviewed for any malaria-like symptom or fever with in past 48 h before study commencement. Armpit digital thermometers were used to measure the axillary temperature. Laboratory technologists conducted testing of finger-prick blood by Care Start™ Malaria *PAN (pLDH)* Ag Rapid Diagnostic Tests (RDTs) [23]. Field data collected by EpiSample were every day sent to the central EPHI data management server and backup data was downloaded from the internet server to a computer every week.

### Statistical analysis of data

Data were cleaned and analyzed using STATA 14 package [24]. Descriptive statistics were used to describe the characteristics of the sample and calculate coverage, use, and access estimates. Point estimates and confidence intervals were analyzed to adjust clustering in the sampling design, with weighting for household and cluster sampling probability [25]. The descriptive statistics and differences in distribution were evaluated using the Chi-square (χ2) test and *P-value < 0.05* was considered statistically significant.

### Definition of terms

#### Asymptomatic malaria

Are cases tested positive by malaria RDTs but were with an axillary temperature of < 37.5 °C as measured by a digital thermometer or/and those who had reported no fever history or no malaria-like symptoms within 48 h before field data collection.

#### Symptomatic malaria

Are cases tested positive by malaria RDTs, and had an axillary temperature of ≥37.5 °C as measured by a digital thermometer or/and had fever history or other malaria-like symptoms within 48 h before field data collection.

## Results

### Characteristics of the study population

This baseline assessment was conducted in twenty malaria elimination targeted Districts, selected from five Regions and one city administration. The Regions are Harar, Oromia, Amhara, Southern Nations Nationalities and Peoples (SNNP), and Tigray Regions; and Dire Dawa city administration. At the total study households, 35,900 participants were initially registered into the ODK program installed in the Smartphone; of which, 29,993 gave axillary temperature for temperature measurement. Out of those who gave axillary temperature, 28,973 participants tested for malaria with RDTs (Table 1).

**Table 1:** Characteristics of study sites and population by Regions in Ethiopia, Oct-Dec 2018

Characteristics		Amhara, N (%)	Dire dawa N (%)	Harari N (%)	Oromia, N (%)	SNNPR, N (%)	Tigray, N (%)	Total, N (%)
**Age in yrs**	< 5	609 (9.6)	145 (12)	65 (6.1)	1245 (13.2)	1533 (12.1)	461 (9.7)	4058 (11.4)
	5–11	940 (14.7)	213 (17.7)	174 (16.4)	1726 (18.3)	3126 (24.7)	903 (19)	7082 (20)
	12–17	679 (10.6)	112 (9.3)	102 (9.6)	1049 (11.1)	1884 (14.9)	569 (12)	4395 (12.4)
	18–59	3699 (58)	667 (55.3)	656 (61.8)	4697 (49.7)	5659 (44.8)	2317 (48.8)	17,695 (49.9)
	> 60	449 (7)	69 (5.7)	64 (6)	729 (7.7)	439 (3.5)	499 (10.5)	2249 (6.3)
**Sex**	Female	3277 (50.6)	673 (53.5)	525 (48.4)	4684 (49.3)	6582 (51.5)	2360 (49.5)	18,101 (50.4)
	Male	3203 (49.4)	586 (46.5)	558 (51.5)	4821 (50.7)	6208 (48.5)	2407 (50.5)	17,783 (49.6)
**Fever**	No	5563 (98.2)	917 (99.2)	997 (96.1)	7861 (99.3)	7798 (76.1)	4091 (97.5)	27,227 (90.8)
	Yes	102 (1.8)	7 (0.8)	41 (3.9)	58 (0.7)	2453 (23.9)	105 (2.5)	2766 (9.2)

The mean age of participants was 25 years(yrs). An age category of 18–59 yrs held the highest proportion (44.8–61.8%) in all the study Regions; while, the age category of > 60 yrs covered the lowest proportion in all Regions. The other age groups covered almost a similar proportion in all settings. Gender data showed almost equal proportion in all study settings, despite a slight increase by female participants in some areas (Table 1).

### Malaria prevalence per regions

The overall prevalence of malaria as detected by RDTs in this survey was 1.17% (339/28983) among the total study participants. A high proportion of malaria infection was reported from Harari 46(4.7%) followed by 87(3.7%) in Kersa, 81(2.7%) in Misrak Badawacho, 27(1.7%) in Kolla Tembien, 32(1.4%) in Habru and 22(1.07%) in Raya Kobo Districts. In half (50%) of the surveyed Districts, the prevalence of malaria was in between greater zero & < 1% by RDTs. Interestingly, four Districts namely Berehet, Sire, Gemechis, and Damboya reported zero prevalence of malaria by the RDTs (Table 2).

**Table 2:** Prevalence of malaria using RDTs by Regions and Districts in Ethiopia, October to December 2018

Regions	Name of Districts	RDT Results		
		Total, n (%)	Positive, n(%)	95% CI for Positives
Amhara	Berehet	531 (100))	0	0.0–0.69
	Bugina	626 (100	1 (0.16)	0.0–0.89
	Raya kobo	2055 (100)	22 (1.07)	0.67–1.62
	Habru	2281 (100)	32 (1.4)	0.96–1.97
	Total	5493 (100)	55 (1)	0.76–1.3
Dire dawa		886 (100)	4 (0.45)	0.12–1.15
Harari		979 (100)	46 (4.7)	3.46–6.22
Oromia	Merti	871 (100)	8 (0.92)	0.4–1.8
	Sire	729 (100)	0	0.0–0.5
	Zeway-dugida	2433 (100)	10 (0.41)	0.2–0.75
	Kersa	2358 (100)	87 (3.69)	2.97–4.53
	Gemechis	1447 (100)	0	0.0–0.25
	Total	7838 (100)	105 (1.34)	1.1–1.62
SNNPR	Dilla-zuria	503 (100)	2 (0.4)	0.05–1.43
	Marako	1816 (100)	1 (0.06)	0.0–0.31
	Misrak-badawacho	2999 (100)	81 (2.7)	2.15–3.35
	Damboya	816 (100)	0	0.0–0.45
	Dore (Hawassa-zuria)	1403 (100)	1 (0.07)	0.0–0.40
	Lanfaro	2129 (100)	1 (0.05)	0.0–0.26
	Total	9666 (100)	86 (0.9)	0.71–1.1
Tigray	Kolla-Tembein	1597 (100)	27 (1.69)	1.12–2.45
	Hawzen	1235 (100)	5 (0.4)	0.13–0.94
	Seharti-Samre	1289 (100)	11 (0.9)	0.43–1.52
	Total	4121 (100)	43 (1.04)	0.76–1.4
Total		28,973 (100)	339 (1.17)	1.05–1.3

CI= $\boldsymbol{P}\pm \boldsymbol{z}\sqrt{\boldsymbol{P}\left(\mathbf{1}-\boldsymbol{P}\right)/\boldsymbol{n}}$; where **z** value for 95% = 1.96, **p** = sample proportion, **n** = sample size, **CI** = confidence interval

### Malaria and socio-demographic characteristics

From a total of 28,983 survey participants who were grouped into five age groups, malaria prevalence was highest among the age group 5–11 yrs (1.8%) followed by 12-17 yrs (1.4%), while malaria prevalence was lowest among the age group of 18–59 yrs (0.9%). Malaria prevalence rate among males (1.4%) was higher than females (1%) (*P-value* < 0.001). Malaria prevalence among pregnant women (1.9%) was higher compared to non-pregnant women (*P-value*: 0.023) (Table 3).

**Table 3:** **Malaria prevalence and sociodemographic characteristics in Ethiopia, October to December 2018**

Characteristics	RDT results			P-value
	Total	Positive	95%CI	
**Age group (in years)**				
< 5	2886 (100)	36 (1.25)	0.87–1.72	0.677
5–11	5963 (100)	107 (1.79)	1.47–2.16	0.044
12–17	3535 (100)	50 (1.41)	1.05–1.86	0.354
18–59	14,703 (100)	125 (0.85)	0.71–1.01	0.251
> 60(Reference)	1886 (100)	21 (1.1)	0.69–1.7	
Total	28,973 (100)	339 (1.17)	1.05–1.30	
**Sex**				
Female (Reference)	15,566 (100)	149 (0.96)	0.81–1.12	
Male	13,407 (100)	190 (1.42)	1.22–1.63	< 0.001
Total	28,973 (100)	339 (1.17)	1.05–1.30	
**Pregnant Women**				
Yes	514 (100)	10 (1.95)	0.94–3.55	0.023
No (Reference)	7590 (100)	58 (0.76)	0.58–0.99	
Don’t Know	36 (100)	0	0.0–9.74	0.988
Total	8140 (100)	68 (0.84)	0.65–1.06	
**Travel History (in the last 3 months), n (%)**				
Yes	418 (100)	7 (1.67)	0.68–3.42	0.274
No	25,617 (100)	283 (1.1)	0.98–1.24	Ref
Total	26,035 (100)	290 (1.1)	0.99–1.25	
**Fever status, n (%)**				
Symptomatic (axillary temp> 37.5oC)	2510 (100)	84 (3.35)	2.68–4.13	< 0.001
Non-symptomatic (axillary temp≤37.5oC)	26,463 (100)	255 (0.96)	0.85–1.09	Ref
Total	28,973 (100)	339 (1.17)	1.05–1.30	
**The altitude of the study area (M), n(%)**				
1000–1500	3095 (100)	65 (2.1)	1.62–2.67	< 0.001
1501–2000	18,834 (100)	167 (0.89)	0.76–1.03	0.043
> 2000	3453 (100)	51 (1.48)	1.1–1.94	Ref
Total	25,382 (100)	283 (1.11)	0.99–1.25	

Among the 2510 symptomatic individuals having axillary temperature > 37.5 °C, only 84(3.35%) were malaria positive while 2426 (96.65%) were malaria negative. There was a significant association between fever and malaria positivity (*P-value* < 0.001) (Table 3). Among the total study participants, malaria prevalence in asymptomatic individuals was 0.88% (255/28973) and in symptomatic individuals was 0.29% (84/28973). Among all malaria positives, 75.2% (255/339) were asymptomatic and 24.8% (84/339) were symptomatic (Table 3).

The highest malaria prevalence was seen at the lower altitude (1501-2000 m) above sea level (ASL), while less prevalence occurred at > 2000 m. There was observed a significant relationship between lower altitude and malaria prevalence (*P-value* < 0.001). The assessment showed travel history (within the last 3 months) had no association (*P-value:* 0.274) with malaria positivity. Most of the malaria positives had no travel history. Of those having travel history within the last 3 months, 98.3% (418) were malaria negative while only 1.7% were malaria positive (Table 3).

Where; **ref**: reference, **CI:** Confidence Interval, **n:** sample size

Among the asymptomatic malaria patients (255), the highest proportion was observed in 18–59 yrs of age, which was 38.4%(98); followed by 29.4%(75) in 5–11 yrs, 16.1%(41) in 12–17 yrs, 10.5%(26) in under 5 yrs, and 5.9%(15) in > 60 yrs of age. This means asymptomatic malaria prevalence was 10.2% (26/255) in under-five children and 89.8% (229/255) in age groups above 5 yrs (Table 3).

## Discussion

**Fig. 2:**
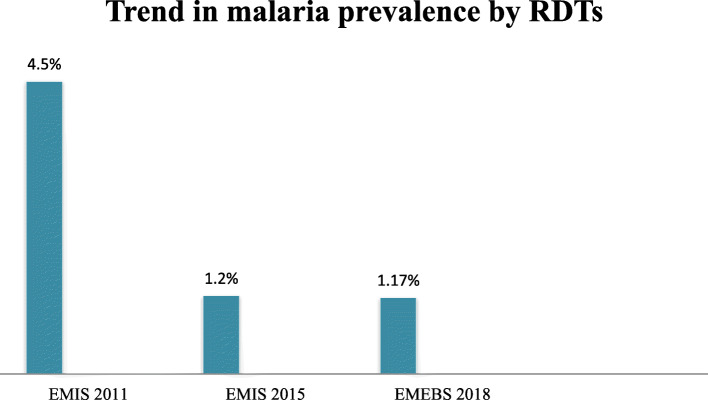
Trends in malaria prevalence by RDTs: Ethiopia MIS 2011, MIS 2015, and the current Malaria elimination baseline survey 2018. Where **EMIS**: Ethiopian malaria indicator survey; **EMEBS**: Ethiopian malaria elimination baseline survey

The current study provided baseline information for the malaria elimination program in the targeted low transmission Districts of Ethiopia. The overall prevalence of malaria by RDT in this survey was 1.17% (339/28973). In this survey, malaria prevalence ranged from zero to 4.7% at the District level by RDTs. In half (50%) of the surveyed Districts, malaria prevalence was above zero and less than 1% by RDTs. This decreasing malaria is supported by the previous Regional level malaria prevalence reports in Ethiopia [12–14] and also by the findings of Ethiopian national-level malaria indicator surveys of 2007, 2011, and 2015 [18–20]. Although the current baseline survey was conducted in the targeted five Regions and in one city administration different from the national malaria indicator surveys (MIS) which were conducted in all nine regions in the country, we tried to compare the current malaria prevalence with previous reports. Figure 2 shows a trend in malaria prevalence among the Ethiopian MIS 2007, MIS 2011, and MIS 2015, and in the present malaria elimination-baseline survey by RDTs. There was a decrease in malaria prevalence by RDTs in the current study (1.17%) compared to the results of MIS 2015 (1.2%), and MIS 2011 (4.5%) (Fig. 2).

**Fig. 3:**
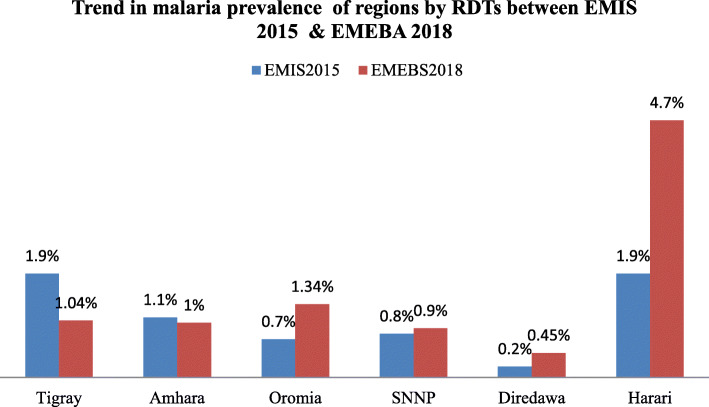
Malaria prevalence among all ages by RDTs in EMIS 2015 and EMEBA 2018. Where, **EMEBS:** Ethiopian malaria elimination baseline survey, **SNNP**: Southern nations, nationalities and peoples’ Region

There were observed changes in malaria prevalence among Regions between MIS 2015 and the current baseline survey. A decrease in malaria prevalence occurred in Tigray and Amhara Regions; while, a little increase in malaria was seen in Oromia, Southern Nations, and Nationalities of Peoples Region (SNNPR) and Dire Dawa City Administration between MIS 2015 and the current baseline survey. Malaria increased from 1.9 to 4.7% in Harari Region between EMIS 2015 and the current survey (Fig. 3). The decrease in malaria prevalence in some Regions shows the strong and continuous malaria intervention approaches in these Regions; while, increased malaria prevalence in other Regions might have occurred due to several reasons such as gaps in malaria control and prevention interventions, changes in climatic conditions and parasitic factors. Another possible reason for the fluctuation of malaria transmission in Ethiopia might be the unstable nature of malaria transmission at diverse geographies of the country. In Ethiopia, there are low malaria cases in a given year and also there may be high malaria reports in another year at a similar set up [26].

Malaria prevalence was higher among the age group 5–11 years (1.8%) followed by 12–17 years (1.4%), while the prevalence rate was low among the age group of 18–59 years (0.9%). However, the difference in malaria prevalence among age groups was not significant. Among the total asymptomatic malaria patients (255), the highest proportion was observed in 18–59 yrs. of age, which was 38.4% (98). Asymptomatic malaria prevalence was 10.2% (26/255) in under-five children and 89.8% (229/255) in age groups above 5 years. This may be so because the older ages might have developed acquired immunity from repeated previous exposures that could block malaria symptom development as reported by other studies [27].

There was a little increase in malaria prevalence among males (1.4%) than females (1%). Males usually stay in outdoor activities more frequently than females and hence are more exposed to mosquito bites. Currently, studies reported that mosquitoes change their behavior from indoor biting to outdoor, or probably new mosquitoes with outdoor biting may have arrived at the study areas [28]. The current malaria prevention and control intervention program gives higher priority for pregnant women and children; however, malaria prevalence among pregnant women (1.9%) was higher when compared to non-pregnant women and those who did not know their pregnancy status. A possible reason for this may be immune suppression and loss of acquired immunity among the pregnant and the hormonal, immunological, and hematological changes during the pregnancy period [29]. High malaria prevalence (59% of all positives) was seen at an altitude of 1501-2000 m ASL, while 23% at 1000-1500 m and 18% at > 2000 m. There was a significant relationship between lower altitude and malaria positivity as stated by another study [30].

In this study, among the 2510 symptomatic individuals having axillary temperature > 37.5^o^C, only 84(3.35%) were malaria positive while 2426 (96.65%) were negative. This may be so because fever is not specific to malaria and it might have been caused by other illnesses. Fever was not fully explained by malaria as reported by other studies in low-resource areas [31]. Although the proportion of infection was lower among symptomatic cases, there was a significant association between fever and malaria positivity.

Asymptomatic malaria cases accounted for 0.88% (255/28973) among the total tested study participants and 75.2% (255/339) among all malaria positives. The asymptomatic malaria cases in low transmission and elimination-targeted areas are potential reservoirs sustaining uninterrupted malaria transmission in the area [32]. Concerning the elimination strategy, WHO remarks that asymptomatic malaria infections should be confirmed by standard diagnostic tests. WHO states that any case confirmed by a diagnostic test is malaria infection whether it is symptomatic or asymptomatic. Active monitoring of malaria infections is necessary for settings where malaria transmission has currently declined and national efforts are underway to achieve malaria elimination.

In Zambia [33], among 3863 household members tested in the elimination target areas, 2.6% of individuals were found positive by either of RDTs, microscopy, or PCR. Of all positives, 47% (48) had sub-patent parasitemia and 85% of sub-patent parasitemia cases were asymptomatic. The study recommended further need of active or reactive case detection approaches to identify more asymptomatic individuals at the community levels during declining malaria. Most countries with low transmission settings and striving to eliminate malaria demonstrated a large proportion of asymptomatic *Plasmodium* infections, particularly submicroscopic parasitemia cases [34–36]. Asymptomatic and subpatent malaria reservoirs can maintain continuous transmission even in low transmission settings unless they are strictly monitored and detected by a highly sensitive diagnostic method, and immediately treated by an effective antimalarial drug [37]. The current study findings forward the necessity of the use of more sensitive molecular diagnostic tools to give a more accurate prevalence of infections in community surveys occurring in low-transmission settings.

The large-scale deployment of RDTs for malaria diagnosis has greatly improved access to confirmatory diagnosis in malaria-endemic countries, contributing to the success of malaria control programs in recent years [38]. RDTs are convenient tests for the screening of a large number of samples in national surveillance studies or other large-scale malaria elimination programs such as mass screening and treatment programs. However, population surveys using traditional diagnostic techniques including microscopy or RDTs may miss submicroscopic infections that are below the detection limit of these tools. Studies in high transmission areas have shown that as many as two-thirds of microscopy-negative patients may have low-grade parasites [39].

False-negative RDT results commonly occur because of professional errors, inconvenient storage situations, histidine-rich protein deletions, low parasitemia, and RDT types with poor performance. The detection capacity of malaria RDTs varies in different transmission settings and false-negative RDT results are common in lower malaria transmission settings [40]. Therefore, highly sensitive and specific malaria diagnostics such as PCR or highly sensitive RDTs are critically needed in community surveys, particularly in low transmission and elimination target settings to explore more precise prevalence estimates [41].

### Strengths and limitations of the study

Although this paper has placed malaria prevalence by RDTs only, this study incorporated a multistage sampling design, used the Smartphones programmed with the ODK based technology for questionnaire interview and household selection, and employed a consolidated efforts of several partner organizations to collect a very large size malaria data. For the quality of data, field-collected data in ODK were daily sent to EPHI online data depository server. Several governmental and non-governmental partner organizations, working on malaria control and elimination, participated in the accomplishment of this baseline survey. Because of lack of reagents, the molecular level prevalence of sub-patent malaria was not done despite a large number of dried blood spots collected in this baseline survey.

## Conclusion

The total malaria prevalence by RDTs in this survey was 1.17%; of which, 0.29% were symptomatic and 0.88% were asymptomatic. The current finding showed a decrease in malaria prevalence compared to the reports of previous malaria surveys in the country and can be used as a baseline for measuring the achievement of ongoing malaria elimination activities. These infections in low transmission and elimination-targeted areas are highly considerable since they may still sustain an uninterrupted malaria transmission in the area. Although RDTs may be convenient serological tests for the screening of a large number of samples in national surveillance studies or other large-scale malaria elimination programs such as mass screening and treatment programs, population surveys using traditional diagnostic techniques including microscopy and/or RDTs may miss submicroscopic infections that are below the detection limit of these tools. Therefore, molecular techniques are more sensitive and thus are more likely to detect sub-patent infectious reservoirs in the elimination settings.

## Data Availability

The datasets used and/or analyzed during the current study are available from the corresponding author on reasonable request or are available in the EPHI data repository

## Abbreviations

ACT: Artemisinin Based Combination Therapy
API: Annual Parasite Incidence
EPHI: Ethiopian Public Health Institute
FMOH: Federal Ministry of Health
HH: Household
HMIS: Health Management Information System
IRS: Indoor Residual Spraying
ITN: Insecticidal Treated Net
LLIN: Long Lasting Insecticidal Treated Net
MIS: Malaria Indicator Survey
NSP: National Strategic Plan
ODK: Open Data Kit
PCR: Polymerase chain reaction
RDT: Rapid Diagnostic Test
